# Low and High Molecular Mass Anthraquinone Derivatives Containing Substituents of Varying Electron Donating Properties: Electrochemical and Spectroelectrochemical Properties

**DOI:** 10.1021/acs.jpcc.5c01028

**Published:** 2025-06-12

**Authors:** Kamil Kotwica, Marek Charyton, Anna Jezuita, Guy Louarn, Grażyna Żukowska, Magdalena Sowa, Nicolas D. Boscher, Adam Proń

**Affiliations:** † Faculty of Chemistry, 49566 Warsaw University of Technology, Warsaw 00-664, Poland; ‡ 87145 Luxembourg Institute of Science and Technology (LIST), Esch-sur-Alzette L-4362, Luxembourg; § Faculty of Science and Technology, 646132 Jan Dlugosz University in Czestochowa, Al. Armii Krajowej 13/15, Czestochowa 42-200, Poland; ∥ Nantes Université, CNRS, 131984 Institut des Matériaux Jean Rouxel (IMN), UMR 6502 CNRS, UMR 6502, Nantes Université, 2 rue de la Houssinière, Nantes 44322, France

## Abstract

Two series of donor–acceptor compounds were investigated, consisting of the same anthraquinone acceptor substituted in either position 1 or in position 2 with donors of varying electron donating properties, namely, phenoxazine (Anth-1-Phenox and Anth-2-Phenox), carbazole (Anth-1-Carb and Anth-2-Carb), or diphenylamine (Anth-1-NPh2 and Anth-2-NPh2). In the negative potential range (vs Fc^+^/Fc) all studied compounds exhibited two reversible redox couples corresponding to two 1e reductions of the anthraquinone unit. These reduction processes showed very little dependence on the donor chemical nature and the positional isomerism, yielding *E*_1/2_(0/–1) in the range from −1.33 V to −1.43 V and *E*_1/2_(−1/–2) in the range from −1.75 V to −1.83 V vs Fc^+^/Fc. To the contrary, their redox potentials of the oxidation processes were strongly dependent on the type of donor, decreasing from 0.82 V for Anth-1-Carb to 0.36 V for Anth-1-Phenox. The corresponding potentials measured for 2-substituted anthraquinones were systematically higher by 90 to 210 mV as compared to their 1-substituted counterparts. Anth-1-Phenox and Anth-2-Phenox showed interesting ambipolar properties, undergoing two consecutive reversible 1e reductions at negative potentials and one reversible 1e oxidation at positive ones. The four remaining compounds did not oxidize reversibly. Quantum chemical (DFT) calculations of the HOMO and LUMO energies as well as the ionization potentials (IPs) and electron affinities (EAs) were in legitimate agreement with the experimental data, in each case reflecting the same trend. Equally good agreement was also found between the experimental UV–vis–NIR spectra and the theoretically calculated transitions. None of the synthesized compounds could be electropolymerized. However, fine-quality thin films of p­(Anth-2-NPh2) could be synthesized and deposited on a suitable substrate starting from the oxidative chemical vapor deposition (oCVD) of (Anth-2-NPh2). The obtained polymer, p­(Anth-2-NPh2), was of an ambipolar nature and showed a relatively narrow band gap (*E*_g_ = 1.52 eV). Combined UV–vis–NIR and Raman spectroelectrochemical investigations revealed that the electrochemical oxidation of this polymer thin film can be considered as a two-step process in which the semiquinone radical cation type of structure is formed in the first step, being then transformed into the diiminium dication form in the second one.

## Graphical abstract

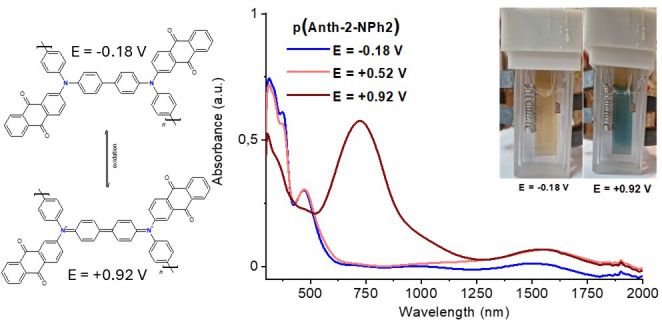

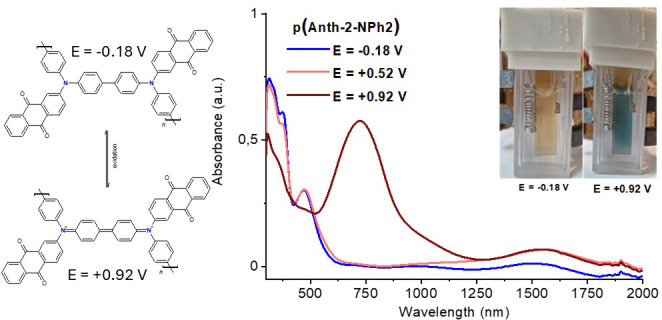

## Introduction

Donor–acceptor (DA) compounds consisting of anthraquinone A units substituted with D units of different electron donating properties have attracted significant research interest in the past decade due to their low band gap, fostering their application in solar energy harvesting.^1^ In addition, anthraquinone-based DA compounds often exhibit interesting photo- and electroluminescent properties that are barely matched by those of other types of organic dyes and luminophores. This involves, among others, the so-called “thermally activated delayed fluorescence” (TADF) effect resulting in a significant improvement of the external quantum efficiency (EQE) values in purely organic light-emitting diodes (OLEDs) through conversion of inactive triplet excitons into singlet ones, which actively participate in the electroluminescence process.^2–5^

Ambipolarity is another feature which makes DA compounds interesting from both basic science and application points of view. Although there exist a plethora of electroactive DA, DAD, and ADA compounds both low- and high-molecular mass, those exhibiting ambipolarity of reversible nature are still very scarce.^6,7^ Our preliminary calculations carried out for anthraquinone derivatives of the DA type unequivocally indicated that they could be considered as potential ambipolar molecules. Moreover, their ambipolarity could be strongly affected not only by adjusting the electron-donating character of the substituent but also by the position of its substitution. Thus, we decided to carry out a systematic study on six anthraquinone-based DA compounds consisting of 9,10-anthraquinone substituted with three types of donors differing in their electron-donating properties, namely, aromatic amine, carbazole, and phenoxazine. With the goal to study the effect of the position of the D groups on the electrochemical properties of the resulting DA compounds, the substitutions were carried out in either position 1 or position 2 of the anthraquinone unit to yield two distinctly different constitutional isomers. It turned out that, using this approach, it was possible to obtain molecules exhibiting practically constant electron affinity (|EA| ∼3.5 eV) and ionization potential (IP) varying from ∼5.10 eV to ∼5.80 eV. Moreover, two compounds of the lowest energy gap exhibited almost perfectly reversible ambipolarity, as probed by cyclic voltammetry. Such studies, never conducted before for anthraquinone derivatives, not only improve the systematic knowledge of these compounds but also have practical significance.

High-molecular-mass DA compounds containing anthraquinone acceptor units may exhibit extraordinary spectroelectrochemical properties, including electrochromism.^8,9^ Anthraquinone itself is electrochemically active undergoing two consecutive reversible one-electron reductions which lead to a radical anion (AQ^•–^) in the first step and to a spinless dianion (AQ2^–^) in the second one.^10^ If an anthraquinone unit is combined in one polymeric chain with an appropriate donor capable of undergoing a reversible oxidation, it can yield a low-band gap polymer assuring stable electrochromism over a large spectral range,^8,9,11^ including its near-infrared part.^12^ Thus, we were tempted to electropolymerize all six anthraquinone-based DA compounds described in this article, albeit without success. We succeeded, however, in polymerizing one of these compounds, namely, anthraquinone substituted with diphenylamine group using a modification of the oxidative chemical vapor deposition method. The obtained fine-quality thin polymer films deposited on a conductive transparent substrate were almost ideally suited for detailed UV–vis–NIR and Raman spectroelectrochemical investigations, which could shine light on the mechanism of redox processes leading to spectral changes in the studied polymer. Raman spectroelectrochemistry is especially well suited for this purpose since these processes inevitably lead to various modifications of selected force constants in the macromolecule as well as in alteration of the sequence of its bonds. All of these changes are reflected in Raman spectra recorded at different working electrode potentials. Despite their strong advantages, the use of combined UV–vis–NIR and Raman spectroelectrochemical methods in the studies of electroactive DA polymers is rather scarce, although few examples have been reported in the literature.^13–15^ Here, we extend this domain of spectroelectrochemistry to anthraquinone-based polymers.

## Experimental Methods

### Synthesis of DA Compounds

The six anthraquinone-based DA compounds were synthesized through a condensation reaction between 1-bromoanthraquinone (or 2-bromoanthraquinone) and the donor compound (diphenylamine or carbazole or phenoxazine) in the presence of tris­(dibenzylideneacetone)­dipalladium(0) as a catalyst. The detailed synthetic procedure can be found in Supporting Information together with a full spectroscopic characterization of the obtained compounds.

### Cyclic Voltammetry

Cyclic voltammograms of the anthraquinone-based DA compounds were recorded by using an Autolab potentiostat Eco Chimie. The electrolytic solution consisted of 0.1 M Bu_4_NBF_4_ dissolved in dichloromethane (DCM). Small amounts of the studied anthraquinone-based DA compound (concentration of 10^–3^ M) were added to it prior to the measurement. Glassy carbon disk was used as the working electrode, Ag/0.1 M AgNO_3_ in CH_3_CN as the reference electrode, and platinum wire as the counter electrode.

### DFT Calculations

Quantum chemical calculations were performed to predict the electron and optical properties of the analyzed molecules. For each studied system, an optimization without any symmetry constraints was carried out (in vacuum and in dichloromethane) with the use of the Gaussian16 package.^16^ Calculations were performed using the DFT method at B3LYP functional,^17,18^ with a 6–311++*g*(d,p) basis set at the ground-state molecular geometries. Solvent effect of dichloromethane was calculated applying of the polarizable continuum model (PCM).^19,20^ The SCF convergence criterion was equal to 10^–8^ Hartrees in convergence on the RMS density matrix and 10^–6^ Hartrees in convergence in energy change. The presented structures correspond to the minima on the potential energy surface, without imaginary frequencies. The optical properties (the oscillator strengths and energies of the vertical singlet excitations) were determined using the time-dependent DFT (TD-DFT) formalism^21^ at the same level of theory. Vertical ionization potentials (IPs) and electron affinities (EAs) were estimated from the differences in the total energies of the neutral molecules and respective ion radicals (cations or anions).

### Oxidative Chemical Vapor Deposition of Anthraquinone-Derived DA Polymers

The oCVD experiments were performed in a custom-built oCVD reactor equipped with two evaporators (Scheme S1 in the Supporting Information). The evaporators were loaded with several tens of milligrams of the selected anthraquinone-derived DA monomer and several hundreds of milligrams of iron­(III) chloride (FeCl_3_). Preliminary investigations enabled determination of the sublimation temperature under reduced pressure (10^–3^ mbar) of the selected anthraquinone-derived DA monomer. The exact deposition conditions, including the sublimation temperatures and sublimed amounts, are reported in Table S2.

### Characterization of the Anthraquinone-Derived DA Monomers and Polymers

Laser desorption/ionization high-resolution mass spectrometry (LDI-HRMS) was performed directly on the as-deposited thin films using an LTQ/Orbitrap Elite Hybrid Linear Ion Trap-Orbitrap mass spectrometer from Thermo Scientific coupled with an AP-MALDI (ng) UHR source from MassTech Inc., equipped with a 355 nm Nd:YAG laser.

Cyclic Voltammetry and UV–vis–NIR and Raman Spectroelectrochemistry of p­(Anth-2-NPh2) obtained by Oxidative Chemical Vapor Deposition: For cyclic voltammetry investigations, a thin film of p­(Anth-2-NPh2) was deposited on a FTO electrode and the measurements were carried out in the same 0.1 M Bu_4_NBF_4_/DCM electrolyte as in the case of DA compounds.

Layers studied by UV–vis–NIR spectroelectrochemistry and Raman spectroscopy were also deposited on a FTO electrode. In both experiments, potentials were being changed stepwise and the spectra were recorded after a decrease of the current to zero or to a negligible value. UV–vis–NIR spectra were recorded on a Cary 5000 (Varian) spectrometer, whereas Raman spectra were registered using a Nicolet Almega spectrometer (λ_exc._ = 780 nm).

## Results and Discussion

### Electrochemical and Spectroscopic Properties of Monomeric Anthraquinone Derivatives

The studied anthraquinone derivatives are depicted in Chart 1. Only 1-(10H-phenoxazin-10-yl)­anthraquinone (Anth-1-Phenox) is a new compound, the remaining five were previously reported: 2-(diphenylamino)­anthraquinone (Anth-2-NPh2);^22,23^ 2-(carbazol-9-yl)­anthraquinone (Anth-2-Carb);^24^ 2-(10H-phenoxazin-10 yl)­anthraquinone (Anth-2-Phenox);^4^ 1-(diphenylamino)­anthraquinone (Anth-1-NPh2);^25,26^ 1-(carbazol-9-yl)­anthraquinone (Anth-1-Carb),^25^ serving mainly as electroluminophores. Detailed preparation procedures of the investigated compounds can be found in the Supporting Information.

**Chart 1:**
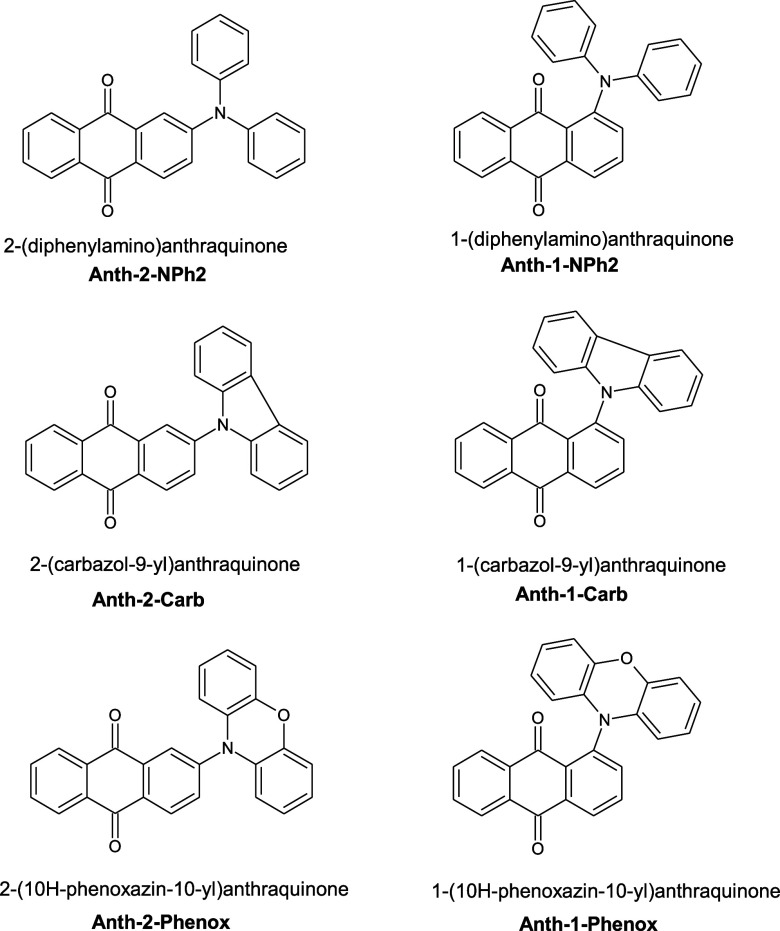
Anthraquinone derivatives containing substituents of varying electron-donating properties. In each case, two positional isomers were investigated.

Cyclic voltammograms of all six anthraquinone derivatives are presented in Figure 1. At negative potentials, they all show two reversible redox couples ascribed to the reduction of the anthraquinone moiety to a radical anion (0/–1) in the first step and to a spinless dianion (−1/–2) in the second one.^10^ All electrochemical data derived from cyclic voltammetry are listed in Table 1. As follows from these results, the formal redox potential of the first redox couple (*E*_1/2_ (0/–1)) is very weakly dependent on the type of the donor substituent and on the substitution position (1 vs 2). The values of *E*_1/2_ (0/–1) determined for carbazole- and phenoxazine-substituted anthraquinone are very close to that measured in DMF for unsubstituted anthraquinone (−1.32 V vs Fc^+^/Fc).^10^ Diphenyl amine-substituted anthraquinone is somehow more difficult to reduce showing higher |*E*_1/2_ (0/–1)| by over 100 mV. In general, substitution with strongly electron-donating groups (e.g., –NH_2_) shifts |*E*_1/2_ (0/–1)| of anthraquinone-based DA compounds to values higher by 140–180 mV, this increase being more pronounced for derivatives being substituted in position 2.^10^ A very weak effect of either the substituent chemical nature or its position on the first reduction process in phenoxazine or carbazole derivatives of anthraquinone seems to indicate that the distribution of the LUMO frontier orbitals is, in the case of these compounds, limited to the anthraquinone moiety. To the contrary, a measurable increase of |*E*_1/2_ (0/–1)| of diphenyl-substituted anthraquinone as compared to the case of anthraquinone can be considered as an indication of some extension of the LUMO frontiers orbitals to the donor part of the molecule. This is confirmed by DFT calculations (*vide infra*).

**Figure 1:**
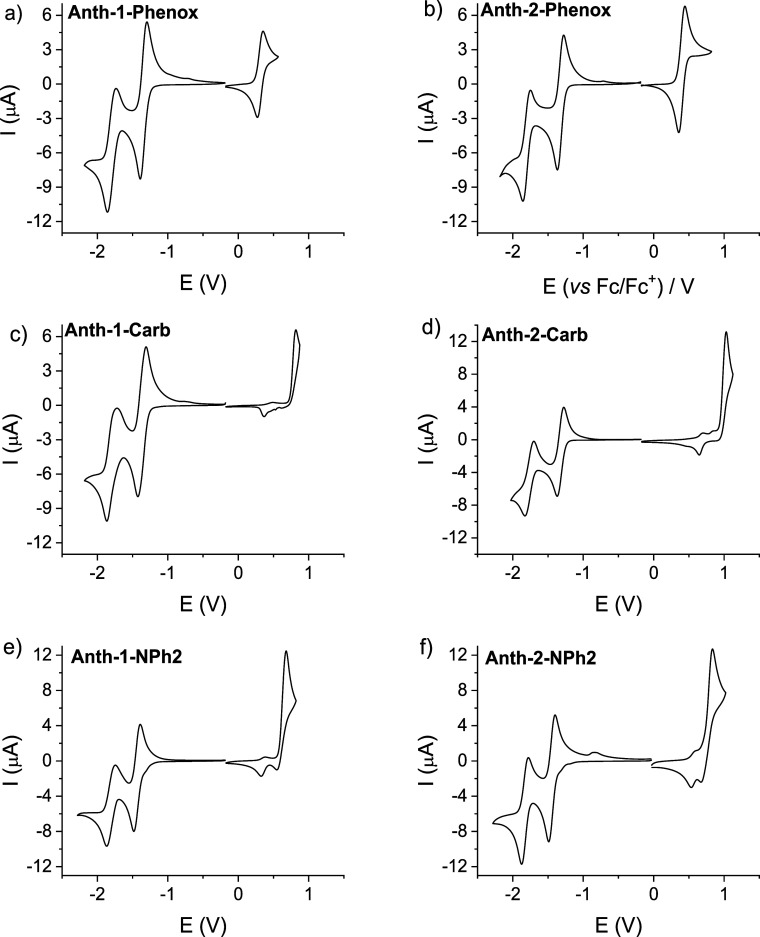
Cyclic voltammograms of: Anth-1-Phenox (a), Anth-2-Phenox (b), Anth-1-Carb (c), Anth-2-Carb (d), Anth-1-NPh2 (e), Anth-2-NPh2 (f); 0.1 M Bu_4_NBF_4_ in DCM; scan rate 50 mV/s; potential vs Fc^+^/Fc.

**Table 1:** Redox Potentials of the Six Studied Anthraquinone-Based DA Compounds (Potential vs Fc^+^/Fc)

	*E*_1/2_(0/–1)	*E*_1/2_(−1/–2)	*E*_cathodic peak_	*E*_anodic peak_	*E*_red_ onset^a^	*E*_ox_ onset^b^
Anth-1-NPh2	–1.43	–1.80	–1.47	0.69	–1.35	0.58
Anth-2-NPh2	–1.44	–1.82	–1.49	0.85	–1.36	0.72
Anth-1-Carb	–1.36	–1.79	–1.42	0.82	–1.27	0.74
Anth-2-Carb	–1.33	–1.75	–1.37	1.04	–1.25	0.95
Anth-1-Phenox	–1.34	–1.79	–1.38	0.36	–1.26	0.25
Anth-2-Phenox	–1.32	–1.80	–1.36	0.45	–1.25	0.33

a First cathodic peak onset potential.

b Anodic peak onset potential.

Phenoxazine, itself, is readily oxidizable, undergoing a reversible one-step, 1e oxidation at relatively low potentials (*E*_1/2_ (0/+1) = +022 V vs Fc^+^/Fc).^27^ Upon binding to strongly electron-accepting anthraquinone, it does not lose its electrochemical reversibility, but its oxidation becomes more difficult, as manifested by an increase of *E*_1/2_ (0/+1) to 0.32 and 0.40 V for Anth-1-Phenox and Anth-2-Phenox, respectively. Electrochemical oxidation of Anth-1-Carb, Anth-2-Carb, Anth-1-NPh2, and Anth-1-NPh2 which occurs at significantly higher potentials is not reversible and, contrary to our expectations, does not lead to electropolymerization. A clear trend can be observed in the investigated series of compounds: the oxidation of anthraquinone derivatives substituted in position 1 is systematically easier than those substituted in position 2 (Figure 1 and Table 1). A similar trend was reported for positional isomers of phenoxazine-substituted *N*-hexylacridone.^27^

It is tempting to rationalize the trend described above by analyzing the distribution of the frontier orbitals in molecules of the studied derivatives (Figure 2). In the case of Anth-1-Phenox, Anth-2-Phenox, Anth-1-Carb, and Anth-2-Carb, the LUMO frontier orbitals are strictly localized on the anthraquinone part of the molecule with no contribution from the donor substituent. As a result, their first reduction step, involving addition of an extra electron to the empty LUMO, should occur at the same or very similar electrode potential as in the case of unsubstituted anthraquinone, which is indeed observed experimentally. LUMOs of Anth-1-NPh2 and Anth-2-NPh2 are somehow extended to the amine nitrogen, manifesting a small contribution of the donor substituent to the LUMO energy. Electrochemical consequence of this contribution is manifested in an increase of the value of |*E*_1/2_ (0/–1)| by 70–110 mV as compared to unsubstituted anthraquinone and the other four derivatives investigated.

**Figure 2:**
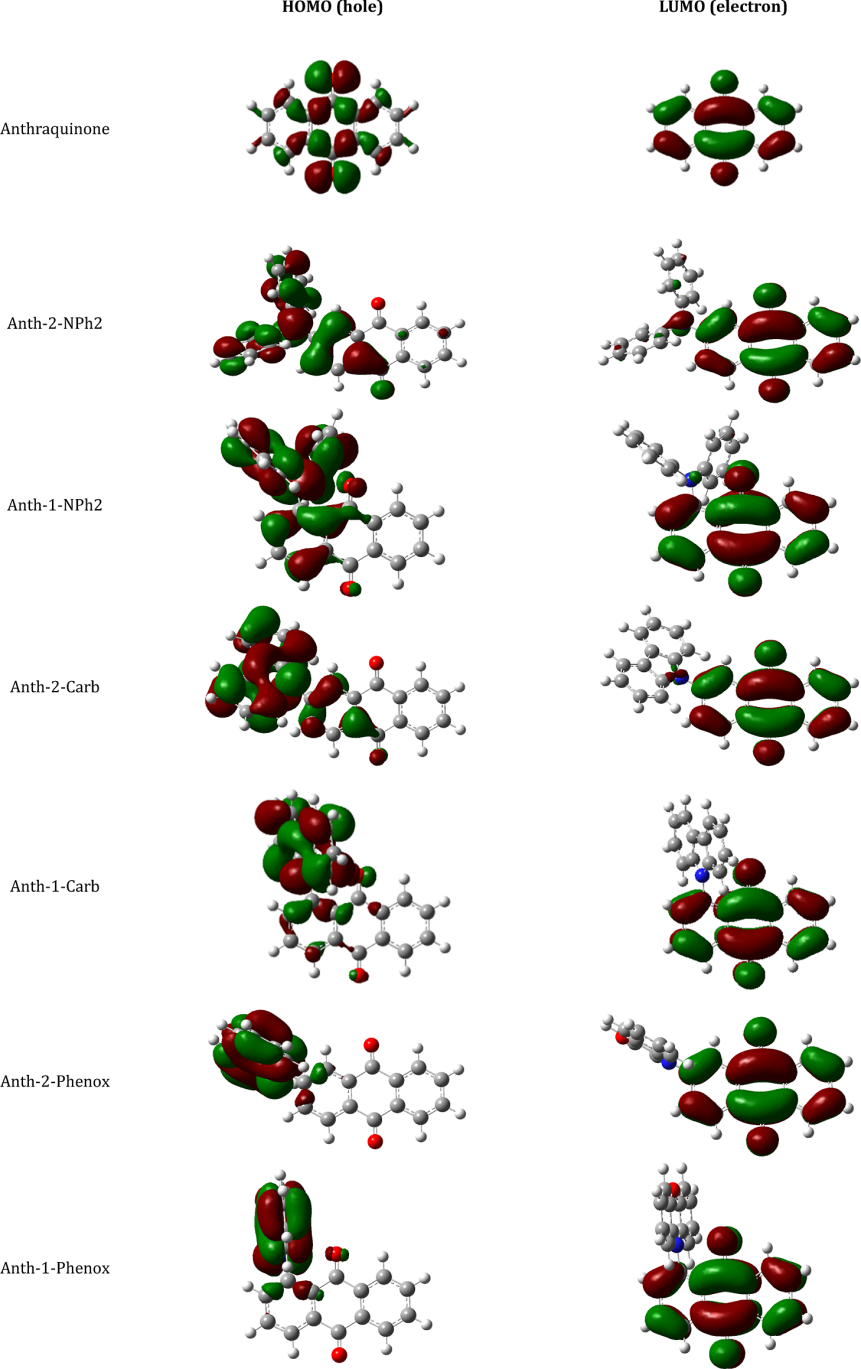
Distribution of HOMO and LUMO in molecules of the studied anthraquinone derivatives.

As already noted, the oxidation potential of the investigated derivatives is not only dependent on the donor substituent chemical nature but also systematically lower for isomers substituted in position 1 as compared to those substituted in position 2. This trend is in agreement with the changes in ionization potentials (IPs) derived from DFT calculations. In all six cases, the HOMO is not limited to the donor part of the molecule, but it is extended to the acceptor (anthraquinone) one, the oxidation potential values reflecting the amount of this extension.

In Table 2, the ionization potential (IP) and electron affinity (EA) values, derived from the electrochemical data, are collected and compared with the corresponding values obtained from theoretical calculations (DFT). As expected, the same trends were found for the experimental and theoretical values; however, the experimentally determined IPs were systematically lower by 0.15–0.30 eV as compared to theoretically calculated ones. To the contrary, the experimental |EA| values were higher than those theoretically calculated by 0.20–0.30 eV.

**Table 2:** Electron Affinity (EA) and Ionization Potential (IP) Values Derived from Cyclic Voltammetry^a^ (Denoted as exp) and from DFT Calculations

compound		HOMO	LUMO	*E*_g_	DFT calc. vs electrochem	
					IP	|EA|
anthraquinone	vacuum	–7.40	–3.19	4.21	9.09	1.60
	DCM	–7.54	–3.28	4.26	7.64	3.24
Anth-1- NPh2	vacuum	–5.54	–3.02	2.52		
	DCM	–5.61	–3.16	2.44	5.59	3.14
	exp				5.38	3.45
Anth-2- NPh2	vacuum	–5.73	–2.95	2.78		
	DCM	–5.78	–3.15	2.64	5.76	3.13
	exp				5.52	3.44
Anth-1-Carb	vacuum	–5.71	–3.29	2.42		
	DCM	–5.86	–3.36	2.50	5.87	3.29
	exp				5.54	3.53
Anth-2-Carb	vacuum	–5.94	–3.28	2.66		
	DCM	–5.98	–3.34	2.64	5.97	3.31
	exp				5.75	3.55
Anth-1-Phenox	vacuum	–4.99	–3.34	1.65		
	DCM	–5.17	–3.62	1.55	5.20	3.31
	exp				5.05	3.54
Anth-2-Phenox	vacuum	–5.22	–3.38	1.84		
	DCM	–5.30	–3.39	1.90	5.33	3.35
	exp				5.13	3.55

a Calculated using the following equations: |EA| = |e|(*E*(0/–1)_onset_ + 4.8), IP = |e|(*E*_ox onset_ + 4.8) [eV] (Table 1).^28^

Absorption spectra of the studied derivatives are comparatively presented in Figure 3. They all show several bands in the visible and UV spectral ranges, i.e., features common for fused aromatic compounds which additionally contain chromophore groups such as carbonyl, aromatic amine, carbazole, etc. (in the study by Rodionova et al.^29^ and supporting information in the works of Zhao X and Zhao J and Huang et al.^4,24^ ). For Anth-1-NPh2, an excellent agreement can be found between the electrochemical band gap (*E*_gel._) and the optical one (*E*_gopt._), whereas in the case of Anth-2-NPh2, this agreement is fair, as clearly seen in Table 3, indirectly indicating that the least energetic optical transition in these two compounds is of the HOMO to LUMO character. This hypothesis is fully corroborated by DFT calculations since the least energetic transition (70% H →L) in these compounds is characterized by a measurable oscillator strength (see Table S1 in the Supporting Information). In the case of other derivatives, the calculated oscillator strengths of the H → L transitions are zero or close to zero, rendering this transition optically undetectable. As a result, large differences are found between the values of *E*_gel_ and *E*_gopt_, the latter representing transitions of higher than H → L energies.

**Figure 3:**
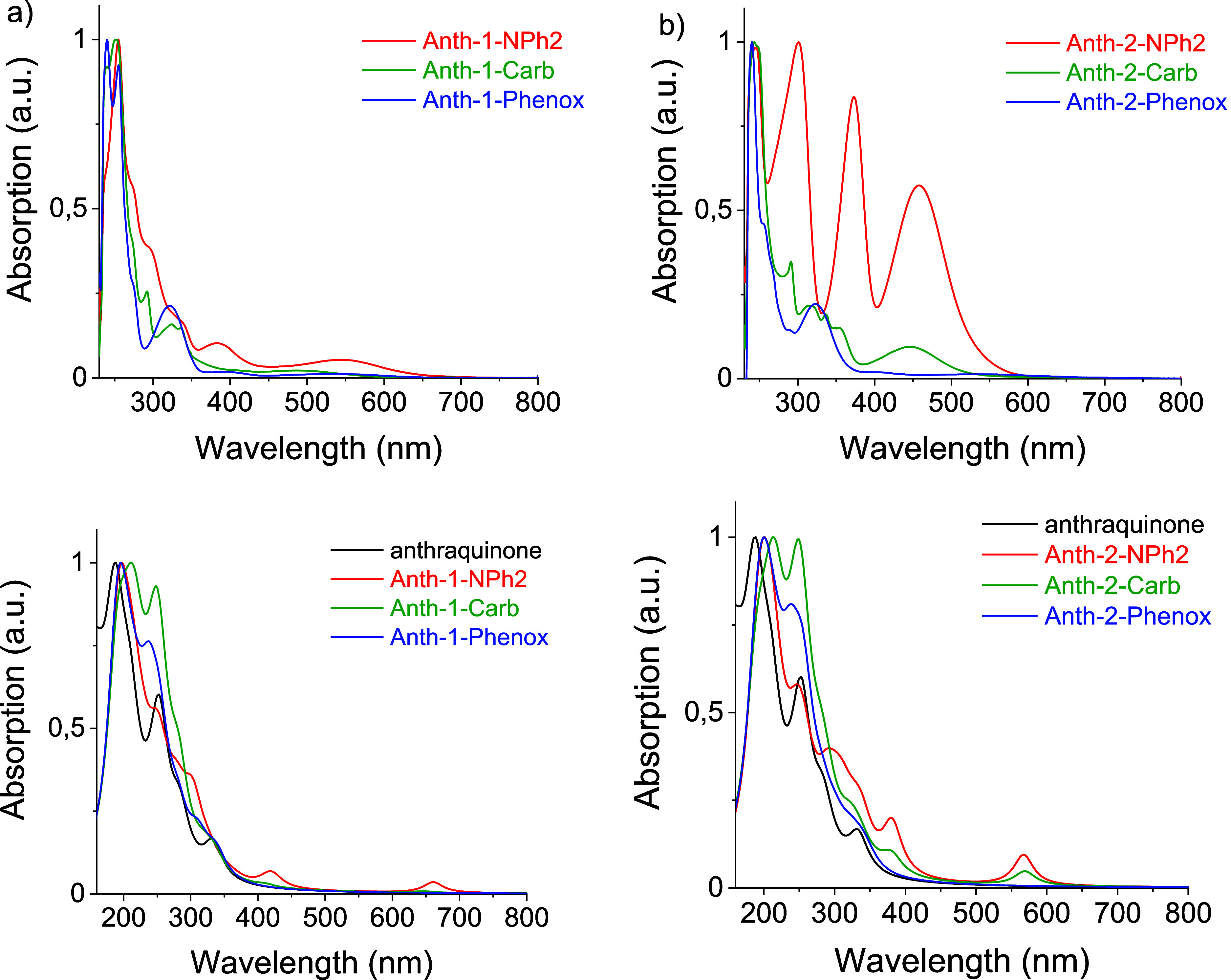
Absorption spectra of anthraquinone derivative solutions in DCM: (a) isomers substituted in position 1; (b) isomers substituted in position 2; top: experimental measurements; bottom: DFT calculation results.

**Table 3:** Spectroscopic Data Together with Optical (*E*_gopt._) and Electrochemical (*E*_gel._) Band Gaps

compound	absorption maxima (nm)							*E*_gopt._ (eV)	*E*_gel._^a^ (eV)
Anth-1-NPh2	254	276	294	380	538			1.91	1.93
Anth-2-NPh2	245	301	373	458				2.33	2.08
Anth-1-Carb	241	251	296	330	341			3.42	2.01
Anth-2-Carb	243	247	291	314	336	353	446	2.36	2.20
Anth-1-Phenox	241	255	271	321	388			3.46	1.51
Anth-2-Phenox	241	257	266	289	326	535		1.91	1.58

a *E*_gel._ = IP–|EA| from cyclic voltammetry (see Tables 1 and 2).

To summarize this first section of the paper, although exhibiting similar reduction potentials, the studied anthraquinone derivatives markedly differ in their electrochemical band gap (*E*_gel._) values due to different capabilities of being oxidized. In particular, the anthraquinone derivatives containing phenoxazine (Anth-1-Phenox and Anth-2-Phenox) are characterized by the smallest *E*_gel._ values, providing at the same time reversibility of two reduction and one oxidation processes. Oxidation of the remaining four derivatives (Anth-1-NPh2, Anth-2-NPh2, Anth-1-Carb, and Anth-2-Carb) occurs at significantly higher potentials and is not reversible. Surprisingly, it does not lead to electrochemical polymerization.

### Electrochemical and Spectroelectrochemical Properties of Polymeric Anthraquinone Derivatives

As an alternative to electropolymerization, oxidative chemical vapor deposition (oCVD) was applied with success to the preparation of polymeric thin films from Anth-2-NPh2. Polymerizations of other monomers via oCVD deposition did not lead to strictly reproducible results. The success of this technique in the case of Anth-2-NPh2 resulted from its capability of undergoing the chemical oxidative polymerization as confirmed in the literature for solution-based methods^22,23^ and its thermal stability in the temperature range of 120 to 210 °C.^23^ oCVD was previously successfully implemented for the oxidative polymerization of polycyclic heterocyclic compounds such as porphyrins^30^ and diketopyrrolopyrroles.^31^ The oCVD reaction of Anth-2- NPh2 was performed at a pressure of 10^–3^ mbar in a custom-made vacuum reactor equipped with two low-temperature evaporation sources (see Scheme S1 in Supporting Information).^32^ The two low-temperature evaporation crucibles were loaded with Anth-2- NPh2 and iron­(III) chloride (FeCl_3_) and heated to 205 and 150 °C, respectively, to sublime the two compounds toward a heated (150 °C) substrate. FeCl_3_ was selected as an oxidant due to its ability to promote the oxidative polymerization of polycyclic heterocyclic compounds in oCVD.^30,32^ To ensure the effective oxidative coupling of the monomer, an excess of the oxidant was sublimed with a molar ratio of oxidant to monomer of 28.

Laser desorption ionization high-resolution mass spectrometry (LDI-HRMS) analysis directly performed on the as-deposited oCVD p­(Ant-2-NPh2) thin film reveals the presence of dimeric and trimeric species (Figure S1), confirming the successful oxidative polymerization of the Anth-2- NPh2 monomer via oCVD. It should be emphasized that LDI-HRMS analysis does not provide an exhaustive view of the mass distribution of the formed polymer thin films and that the intensities related to the different species detected are not directly related to their abundance. Yet, the insolubility of the anthraquinone-derived DA polymer thin films prohibits size exclusion chromatography (SEC) analysis and consequently no detailed molecular mass distribution can be determined. Surprisingly, LDI-HRMS indicates the inclusion of additional diphenylamine moieties into the dimeric and trimeric species (Figure S1a). This alteration from the initial monomer structure is ascribed to the LDI-HRMS analysis and to the relatively strong absorption of the laser light by the polymer and the absence of a matrix. Indeed, similar alteration was observed during LDI-HRMS analysis of the Ant-2-NPh2 monomer (Figure S1b). In high-resolution mass spectrometry of the monomer, where the sample was excited using other ionization methods such as Atmospheric Pressure Chemical Ionization (APCI) (Figure S2a) or Electron Ionization (EI) (Figure S2b), only intact monomer units were detected and no masses corresponding to altered monomer species, i.e., monomer combined with an additional diphenylamine unit, were detected.

One of the most important assets of oCVD is its ability to deposit conjugated polymers directly in thin film forms to readily integrate them in devices and exploit their functional properties.^30,31^ p­(Ant-2-NPh2) was deposited onto various substrates, including microscope glass slides (Figure 4a) and FTO-coated glass substrates. The ca. 100 nm thick p­(Ant-2-NPh2) thin film exhibits a yellowish coloration that slightly differs from the orangish color of the reference sublimed s­(Ant-2-NPh2) thin film prepared from the sublimation of Ant-2-NPh2 in the absence of FeCl_3_. The slight color change is due to a bathochromic shift of the main band situated in the visible region from 461 nm in s­(Ant-2-NPh2) to 480 nm in p­(Ant-2-NPh2) and to the significantly increased absorbance at a high wavelength. The absorbance increase extending up to the near-infrared (NIR) region is attributed to the formation of a conjugated polymer, indicating enhanced electron delocalization along the polymer backbone.

**Figure 4:**
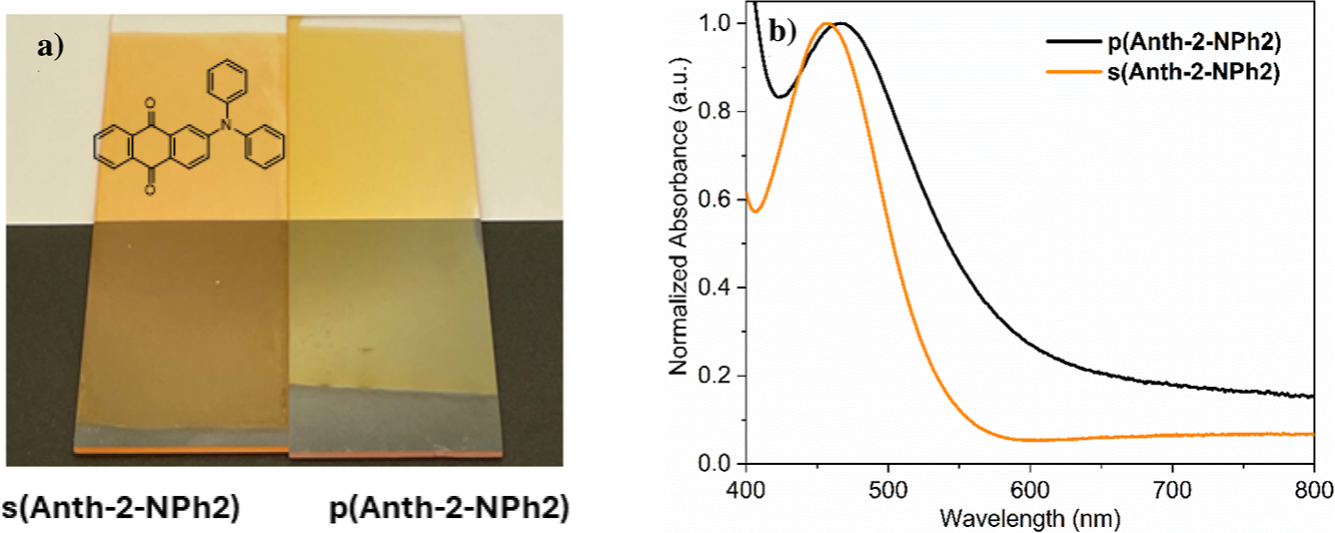
(a) Optical images and (b) normalized absorption spectra in the visible region of the reference sublimed s­(Ant-2NPh2) and oCVD p­(Ant-2-NPh2) thin films prepared on a microscope glass slide from Anth-2-NPh2.

p­(anth-2-NPh2), already reported by Wu et al.,^22^ was simultaneously synthesized and deposited in the form of uniform thin films that strongly adhered to the substrate patterned with platinum or ITO electrodes, thus enabling detailed UV–vis–NIR and Raman spectroelectrochemical investigations. p­(Anth-2-NPh2) is electrochemically active in both the oxidation and reduction modes, as clearly manifested in its cyclic voltammogram (Figure 5). The polymerization has a rather small effect on the reduction process since the value of *E*_red.onset_ determined for p­(Anth-2-NPh2) is equal to −1.29 V vs Fc^+^/Fc, a value which is higher by 0.07 V only as compared to the case of its monomer (Anth-2-NPh2), whereas the cathodic peak potential (*E*_cathodic peak_ = −1.65 V) is lowered by 0.16 V as compared to that of the monomer. Polymerization has a more pronounced effect on the oxidation potential since the anodic peak potential (*E*_anodic peak_ = 0.66 V) of p­(Anth-2-NPh2) is downshifted by 0.36 V with respect to that of Anth-2-NPh2. The anodic peak onset potential (*E*_ox.onset_) is equal to 0.21 V, a value which is lower by 0.41 V than *E*_ox.onset_ of Anth-2-NPh2. As a consequence, its electrochemical band gap is lowered to 1.52 eV.

**Figure 5:**
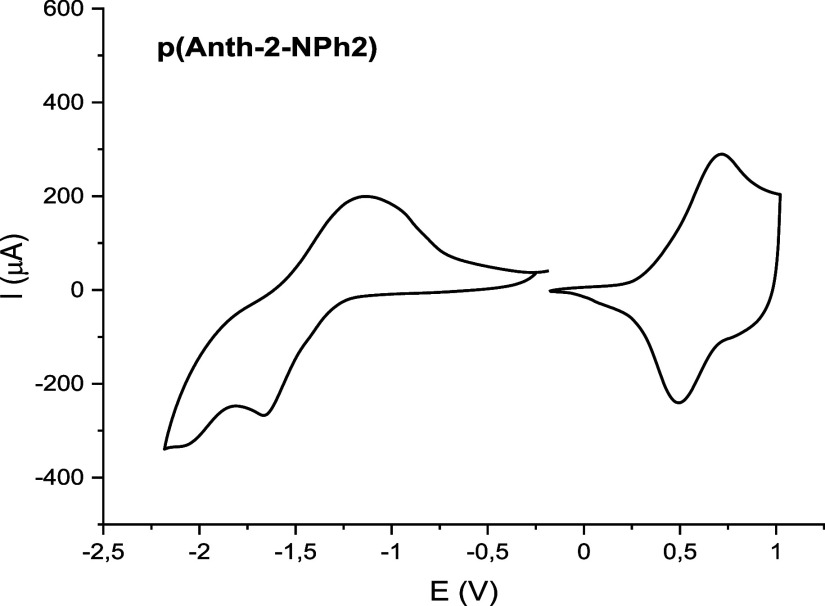
Cyclic voltammograms of a thin film of p­(Anth-2-NPh2) registered at negative and positive potentials; electrolyte 0.1 M Bu_4_NBF_4_ in CH_3_CN; scan rate 50 mV/s; potential vs Fc^+^/Fc.

There are strong differences in the stabilities of the reduced and oxidized states of p­(Anth-2-NPh2) upon electrode potential cycling. When cycled in the negative potential range (−0.20 V to −2.10 V), p­(Anth-2-NPh2) desorbs from the electrode within few cycles, losing its electroactivity. To the contrary, its cycling in the potential range from −0.2 V to +0.8 V shows a very small decrease of its electrochemical activity upon 100 cycles (1–2% as follows from the integration of the CV curves). For a larger number of cycles, this decrease of electroactivity becomes hardly measurable. Similar results are obtained for layers cycled in an extended potential range from −0.2 V to +1.0 V (see Figure S3a,b in the Supporting Information).

As prepared, p­(Anth-2-NPh2) is slightly oxidized, which is manifested by the presence of a weak, very broad band in the NIR part of its UV–vis–NIR spectrum, peaked at ca. 1530 nm. Thus, it must be electrochemically reduced at *E* = −0.18 V vs Fc^+^/Fc to reach the neutral (undoped form) characterized by a UV–vis–NIR spectrum of negligible absorbance in the NIR range (Figure 6).

**Figure 6:**
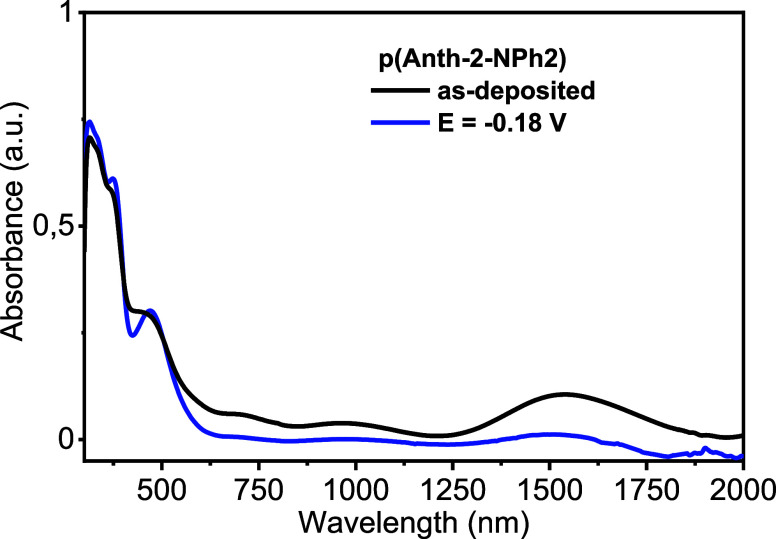
UV–vis–NIR spectrum of as-prepared p­(Anth-2-NPh2) registered in absence of the electrolyte (a) and electrochemically reduced at *E* = −0.18 V vs Fc^+^/Fc (electrolyte 0.1 M Bu_4_NBF_4_).

In Figure 7, the spectra of p­(Anth-2-NPh2) registered at different working electrode potentials are compared. As already stated, *E* = −0.18 V vs Fc^+^/Fc is almost entirely neutral, showing negligible absorbance in the NIR part of the spectrum. The first clearly visible changes in the spectrum start to appear at *E* = 0.52 V. The oxidative doping gives rise to a broad NIR band peaked at ca 1550 nm, whereas the visible part of the spectrum remains essentially unchanged. At 0.62 V, a potential that is very close to that of the anodic peak (*E*_anodic peak_ = 0.66 V), the NIR band becomes dominant, a small increase of the intensity of the band peaked at 480 nm being caused by the increasing background absorption. Further increase of the potential results in profound changes in the UV–vis–NIR spectra of the studied polymer. At E = 0.72 V, the dominant broad NIR band starts to decrease in intensity and a new band peaked at 725 nm concomitantly grows. At *E* = 0.92 V, the NIR band is almost entirely bleached as well as the band at 480 nm present in the spectrum of the neutral and partially doped polymer. Thus, at *E* = 0.92 V, the spectrum shows features of the entirely doped polymer.

**Figure 7:**
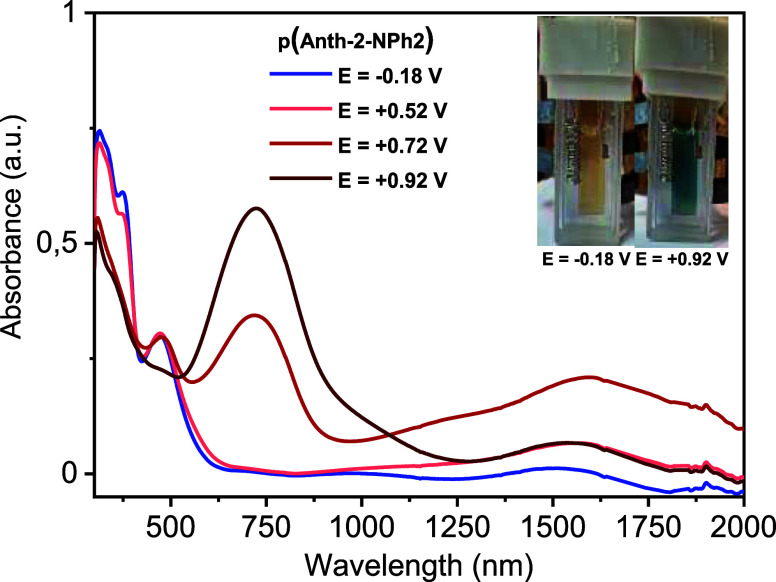
UV–vis–NIR spectra of a thin film of p­(Anth-2-NPh2) registered at different electrode potentials; electrolyte 0.1 M Bu_4_NBF_4_ in CH_3_CN, potential vs Fc^+^/Fc.

High stability of p­(Anth-2-NPh2) upon electrochemical cycling is also reflected by the optical measurements. The UV–vis–NIR spectrum of the neutral polymer prior to cycling is essentially indistinguishable from that measured after 100 cycles. The same applies to the spectrum recorded for the oxidized state (*E* = 1.0 V) within the 1st and the 100th cycle. The colors are also indistinguishable by eye (see Figure S4 in the Supporting Information).

p­(Anth-2-NPh2) can be considered as a special type of donor–acceptor polymer in which the main chain consists of benzidine-type D units *N*-substituted with anthraquinone A units. UV–vis–NIR spectroelectrochemical investigations unequivocally show that oxidative doping involves two spectroscopically distinctly different processes. Considering the aromatic amine nature of the main chain, we are tempted to draw an analogy with oxidative doping of polyaniline in which polarons (semiquinone radical cations) are formed which are then converted into bipolarons.^33^ These processes are depicted in Scheme 1, where two repeating units of the polymer are shown for the sake of clarity.

**Scheme 1:**
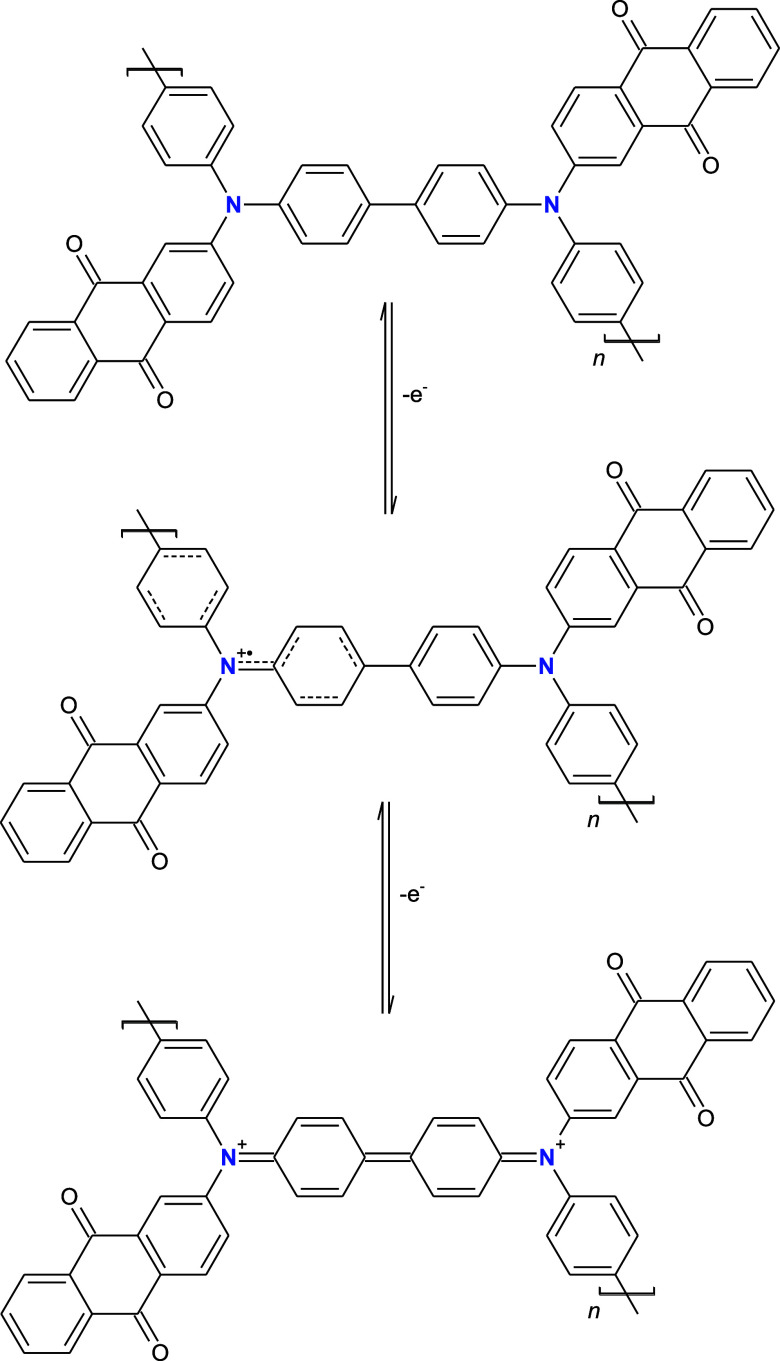
Electrochemical Oxidative Doping of p­(Anth-2-NPh2)

The first process occurring in the potential range from ca. 0.43 to 0.62 V generates radical cations and gives rise to the already mentioned broad NIR band. Further oxidation at potentials above 0.62 V is manifested by bleaching of the NIR band accompanied by the appearance of a new band at 725 nm. Note that it leads to the formation of two iminium cations with a quinoid sequence of the formed double bonds.

Raman spectroscopy seems especially well suited for the confirmation of these types of changes since the oxidation process must involve significant modifications of the force constants of particular bonds and the alteration of the π-bond sequence, i.e., phenomena which inevitably induce Raman spectral changes. In particular, in the cases of spectroelectrochemical investigations of polyaniline^34^ or dendritic oligoarylamines,^35^ this technique allowed for unequivocal differentiation between semiquinone radical, quinoid, and benzoid segments of the macromolecule. It should be noted that the changes in the Raman spectra can be dependent on electrochemically induced changes in the UV–vis–NIR spectra of the polymer (Raman resonance effect). For instance, in the case of Raman spectra registered at *E* = 0.92 V, *E* = 0.82 V, and *E* = 0.72 V, the Raman resonant conditions of the oxidized structure in the polymer are strongly improved. Indeed, the wavelength of the Raman excitation line used in this study (780 nm) is close to the maximum of the absorption band characteristic of the second oxidation state (Figure 7). For the above reasons, we undertook detailed Raman spectroelectrochemical investigations of the electrochemical oxidation of p­(Anth-2-NPh2) supporting these studies by theoretical calculations.

In Figure 8, Raman spectra of p­(Anth-2-NPh2) recorded for increasing electrode potentials are compared. The spectrum registered at *E* = −0.18 V is identical to that measured for the open-circuit potential and can be considered as a characteristic of the neutral form of the polymer. However, its close inspection reveals the presence of two additional bands of low intensity, which cannot be assigned to the vibrations of neutral p­(Anth-2-NPh2). These peaks at ca. 1420 and 1440 cm^–1^ can tentatively be ascribed to minute amounts of slightly oxidized polymer segments whose Raman bands are enhanced in intensity due to the resonance conditions.

**Figure 8:**
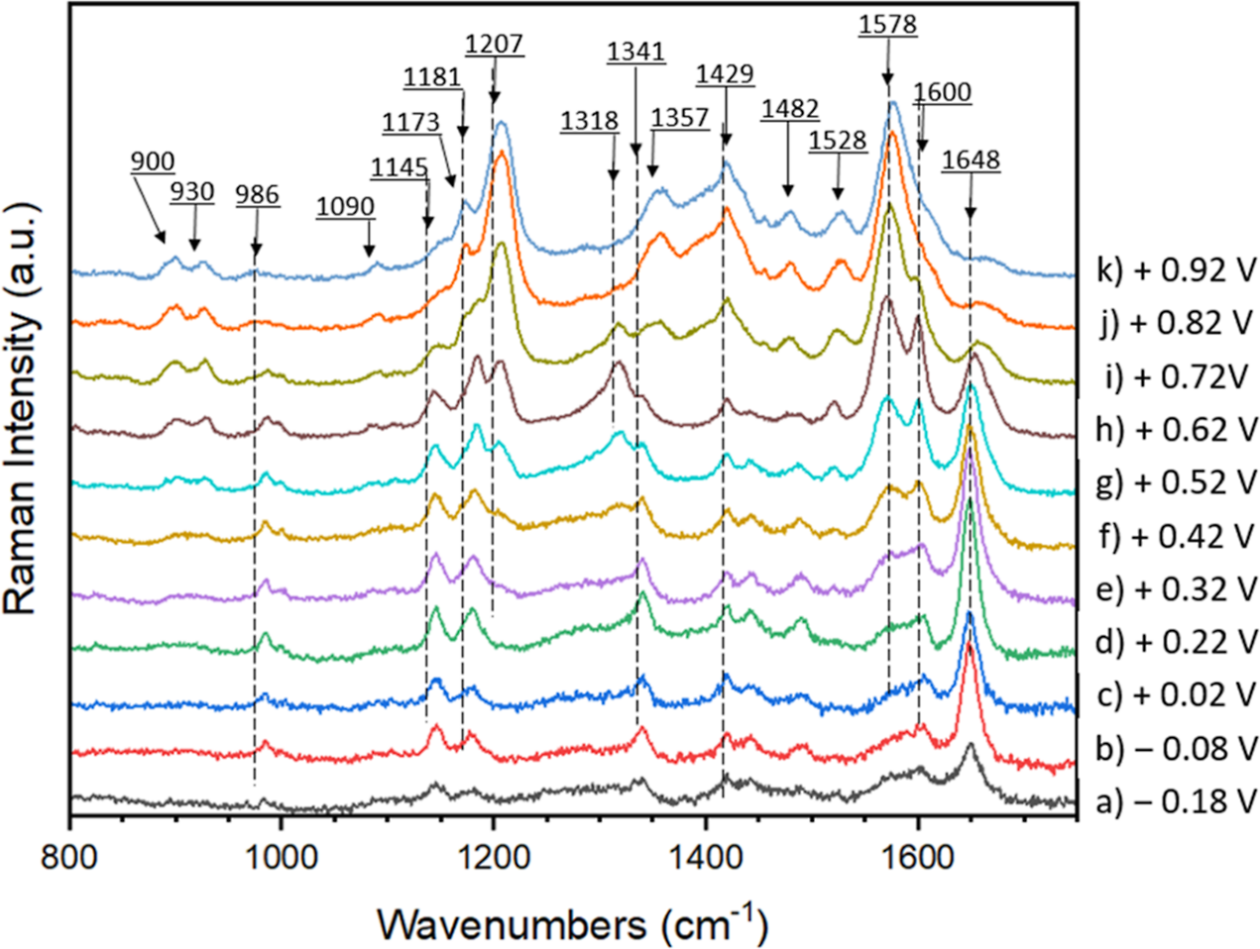
Raman spectra of p­(Anth-2-NPh2) recorded for increasing working electrode potential (electrolyte: 0.1 M Bu_4_NBF_4_ in CH_3_CN, potential vs Fc^+^/Fc).

Oxidation-induced spectral changes start to appear at *E* = 0.22 V, i.e., at a potential which coincides with the anodic peak onset potential (*E*_ox.onset_ = 0.21 V). These spectral modifications also coincide with the appearance and increase of the band at ca. 1550 nm registered in the UV–vis–NIR spectroelectrochemical experiment (compare Figures 6 and 7), which is a characteristic of the first oxidation step. The changes in the Raman spectra seem to indicate that in this first oxidation step, the surplus positive charge density is principally delocalized over the amine site and to a lesser extent over the adjacent aromatic rings, as depicted in Scheme 1.

At potentials exceeding *E* = 0.62 V, profound changes in the Raman spectra can be noticed, involving shifts of selected bands and the appearance of new ones. These changes should be considered as a spectroscopic manifestation of the structural changes in the oxidized polymer chain.

The spectral evolutions are especially profound in three spectral ranges, which are shown in greater detail in Figure 9A–C. In each case, three spectra are compared registered at three different working electrode potentials corresponding to the polymer neutral (*E* = −0.08 V), semioxidized (*E* = 0.62 V), and fully oxidized (*E* = 0.92 V) states, respectively.

**Figure 9:**
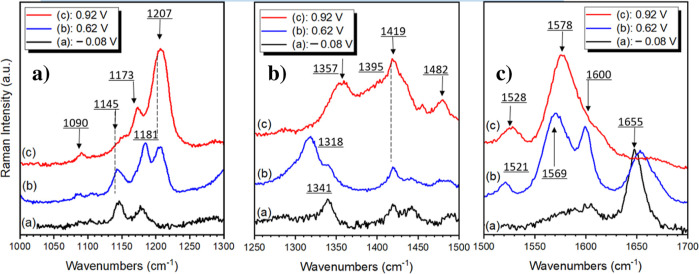
Raman spectra of p­(anth-2-NPh2) registered at three different working electrode potentials: (a) −0.08 V, (b) +0.62 V, and (c) +0.92 V. (A) Spectral range 1000 cm^–1^–1300 cm^–1^, (B) spectral range 1250 cm^–1^–1500 cm^–1^, and (C) 1500 cm^–1^–1700 cm^–1^.

In Figure 9A, the spectral region of the CH bending vibrations is shown. The most pronounced oxidation-induced changes in this spectral range involve the appearance of a new band at 1207 cm^–1^ and simultaneous disappearance of the band at 1145 cm^–1^ upon transformation of the polymer from its neutral through semioxidized to the fully oxidized state. The band at 1181 cm^–1^, present in the neutral polymer as well as in the semioxidized one, shifts to lower wavenumbers upon full oxidation (1173 cm^–1^). These observations indicate that the aromatic rings of the polymer were differently affected during polymer oxidation. The observed spectral changes clearly confirm the proposed two-step oxidation of p­(anth-2-NPh2). They also reflect oxidation-induced modifications of the valence force field and the electronic distribution of aromatic rings.

In the spectral range presented in Figure 9B, Raman bands originating from C=N and C–N stretching vibrations are expected. The oxidation-induced evolution of these Raman bands is very profound in this wavenumber range. In the spectrum registered at *E* = 0.62 V, a new band appears peaked at 1318 cm^–1^, which is tentatively ascribed to the semiquinone radical form (Scheme 1). Upon further electrode polarization to *E* = 0.92 V (fully oxidized state), this band disappears, revealing two new bands 1357 and 1419 cm^–1^ attributed to the formation of diiminium dications (Scheme 1).

Finally, Figure 9C presents this part of the spectral range in which the aromatic C–C and C=O stretching modes of vibration are expected. The C=O stretching mode at 1655 cm^–1^, clearly observed in the Raman spectrum registered at *E* = −0.08 V, slightly shifts to 1670 cm^–1^ for the semioxidized polymer (*E* = 0.62 V) and disappears upon further oxidation (see the spectrum registered at *E* = 0.92 V). The bands at 1569 cm^–1^ and 1600 cm^–1^ present in the spectrum of the semioxidized polymer merge into one band in the spectrum of the fully oxidized polymer with a clear maximum at an intermediate position (1578 cm^–1^).

In order to obtain more exact attribution of the vibrational modes and the polymer structure evolution, we developed a numerical model. The detailed description of the used methodology can be found in ref 36. Here, we only briefly outline it. The calculations of the force field and frequencies were carried out using Fourier’s dynamic matrix. Considering the translational symmetry, the calculations are restricted to only one repeat unit. An internal coordinate system is used to compute the fundamental vibrations of the polymer. The calculation starts from a minimal set of force constants expressed in terms of the internal coordinates. Certain force constants, fairly local, can be transferred from other compounds of an appropriate chemical structure. The parameters are finally adjusted from experimental and calculated frequencies with a least-squares root procedure. The measured and calculated modes together with their vibrational assignments are listed in Table S3 of Supporting Information. The results presented there are largely self-explanatory; therefore, the discussion is confined to the electrochemically induced spectral changes.

To summarize this part of the article, all Raman spectral changes are consistent with the oxidation processes depicted in Scheme 1. In particular, the formation of the semiquinone radical structure in the “partially” oxidized state of p­(Anth-2-NPh2) imposes nonequivalence of nitrogens and some modifications of the aromatic rings adjacent to the positively charged nitrogen. In the fully oxidized state, the nonequivalence of nitrogens is removed through formation of a diiminium dication with a quinoid-type sequence of bonds. All these modifications are clearly confirmed by the observed changes in the Raman spectra recorded at different electrode potentials.

## Conclusions

To summarize, detailed experimental and theoretical studies of six donor–acceptor compounds of the same acceptor (anthraquinone) and donors of varying electron-donating properties (phenoxazine, carbazole, diphenylamine) revealed a strong effect of the donor chemical nature and the type of the positional isomerism (substitution of anthraquinone either in position 1 or 2) on their electrochemical and optical properties. In particular, compounds substituted with phenoxazine (Anth-1-Phenox and Anth-2-Phenox) turned out to be especially interesting, showing low band gaps and ambipolarity manifested by two consecutive 1e reversible reductions and one reversible 1e oxidation. Interestingly, none of the synthesized compounds could be electropolymerized. Interestingly, however, Anth-2-NPh2 could be polymerized via oxidative chemical vapor deposition (oCVD), on a variety of substrates, yielding high-quality thin films of p­(Anth-2-NPh2). In particular, films of this polymer, deposited ITO, or platinum electrodes, exhibited a relatively narrow electrochemical band gap of 1.52 eV. Combined UV–vis–NIR and Raman spectroelectrochemical investigations unequivocally demonstrated a two-step mechanism of this polymer oxidation via the polysemiquinone radical type of structure in the first step to the diiminium dication structure in the second one.

## Supplementary Material



## Notes

Additional experimental details, materials, methods including the scheme of the experimental setup and photographs of the samples, and additional DFT calculations (PDF)
