# Supplementary material for: Low and High Molecular Mass Anthraquinone Derivatives Containing Substituents of Varying Electron Donating Properties: Electrochemical and Spectroelectrochemical Properties
Source: J Phys Chem C Nanomater Interfaces. 2025 Jun 12;129(25):11622–33. doi: 10.1021/acs.jpcc.5c01028 (PMC12207665; doi:10.1021/acs.jpcc.5c01028)
Supplement: Supplementary file 1 [file jp5c01028_si_001.pdf]

Supporting Information

Low and high molecular mass anthraquinone derivatives containing substituents of varying electron donating properties: electrochemical and spectroelectrochemical properties

Kamil Kotwica[a]*, Marek Charyton[b], Anna Jezuita[c], Guy Louarn[d], Grażyna Żukowska[a], Magdalena Sowa[a], Nicolas D. Boscher[b] and Adam Proń[a]

[a]Faculty of Chemistry, Warsaw University of Technology, 00-664 Warsaw, Poland

[b]Luxembourg Institute of Science and Technology (LIST), L-4362 Esch-sur-Alzette, Luxembourg

[c]Faculty of Science and Technology, Jan Dlugosz University in Czestochowa, Al. Armii Krajowej 13/15, 42-200 Czestochowa, Poland

[d]Institut des Matériaux Jean Rouxel (IMN), CNRS, UMR 6502, Nantes Université, 2 rue de la Houssinière, Nantes 44322, , France

Methods

1. **Synthesis :**

General synthesis procedure:
The catalytic system consisted of Tris(dibenzylideneacetone)dipalladium(0) 64 mg (0.07 mmol) with SPhos ligand (43 mg) (0.105 mmol) dissolved in 10 mL of dry toluene under an inert gas atmosphere and stirred for 30 to 45 minutes.
Synthesis of aromatic amines from anthraquinone: The reaction was carried out in a 250 mL Round Bottom Pressure Flask. Sequentially, 1 g of bromoanthraquinone (1-bromoanthraquinone or 2-bromoanthraquinone) (3.48 mmol) and 1 g of sodium tert-butoxide (10.45 mmol) were introduced into the reactor followed by the addition of 70 mL of toluene. In the next step, the catalyst solution ($Pd_2(dba)_3$/SPhos in toluene) was quantitatively transferred, and the flask was rinsed twice with 10 mL of toluene. In the final step, an aromatic amine (diphenylamine or carbazole, or phenoxazine) was added in an amount of 4.18 mmol. After sealing the reactor under an inert gas atmosphere, the reaction was carried out for 24 hours at a temperature of 90°C.
Purification: The post-reaction mixture was extracted using a water-toluene system. The organic phase was dried using anhydrous Na_2SO_4. Purification was performed through column chromatography using CH_2Cl_2 for the initial chromatography, followed by chromatography with a hexane/CH_2Cl_2 system, starting the separation process from pure hexane and gradually increasing the proportion of DCM to 100%.

Ant-2-Phenox 2-(10H-phenoxazin-10-yl)anthraquinone

Yield: from 0.35g to 0.50g (26%-37%) dark purple

^{1}H NMR (400 MHz, $CDCl_3$, δ, ppm): 8,52 (d, J = 6.5 Hz, 1H), 8.37-8.32 (m, 3H), 7.86-7.83 (m, 2H), 7.83-7.79(m, 1H), 6.74 (dtd, J = 9.2, 7.9, 1.5Hz, 4H), 6.67-6.59 (m, 2H), 6.04 (dd, J = 8.0, 1.2Hz, 2H)
^{13}C NMR (100 MHz, δ, ppm): 145.43, 144.48, 136.34, 134.59, 134.49, 133.58, 133.56, 133.29, 132.83, 130.57, 129.39, 127.58, 127.54, 123.51, 122.57, 116.17, 113.94

MS^+:390.1132

Ant-2-Carb 2-(carbazol-9-yl)anthraquinone

Yield: from 0.83g to 0.92g (64%-71%) bright orange

^{1}H NMR (400 MHz, $CDCl_3$, δ, ppm): 8.55 (d, J = 10 Hz, 1H), 8,55 (s, 1H), 8.39-8.31 (m, 2H), 8.13 (ddd, J – 7.9, 1.2, 0.7 Hz, 2H), 8.04 (dd, J = 8.3, 2,2 Hz, 1H), 7.86-7.83 (m, 2H), 7.54 ((dt, J = 8.3, 0.8 Hz, 2H) 7.45 (ddd, J = 8.3, 7.1 1,2 Hz 2H) 7.34 (ddd, J = 8.0, 7.2, 0.9 Hz 2H)

^{13}C NMR (100 MHz, δ, ppm): 182.68, 182.28, 143.58, 139.95, 137.25, 134.57, 134.44, 134.35, 133.66, 131.51, 131.36, 130.33, 129.61, 129.12, 127.51, 127.49, 127.45, 126.58, 124.36, 124.29, 121.25, 120.70, 109.90

MS^+:374.1180

Ant-2-NPh2 2-(diphenylamino)anthraquinone

Yield: from 1.10g to 1.20g (84%-92%), orange

^{1}H NMR (400 MHz, $CDCl_3$, δ, ppm): 8.30-8.26 (m, 1H), 8.23-8.19 (m, 1H), 8.11 (d, J = 9Hz, 1H), 7.77 (d, J = 3.1 Hz, 1H), 7.77-7.69 (m, 2H), 7.40-7.33 (m, 4H), 7.26 (dd, J = 9, 2.6 Hz, 1H) 7.23-7.17 (m, 6H)

^{13}C NMR (100 MHz, δ, ppm): 183.60, 181.78, 153.19, 145.92, 135.06, 134.15, 134.13, 133.73, 133.50, 130.04, 129.32, 127.14, 127.12, 126.46, 125.91, 125.64,0 123.95, 116.59

MS^{+}:375.1261

Ant-1-Phenox 1-(10H-phenoxazin-10-yl)anthraquinone

Yield: from 0.325g to 0.43 g (24%-32%), dark green

^{1}H NMR (400 MHz, $CDCl_3$, δ, ppm): 8.55 (dd, J = 7.8, 1.4 Hz, 1H), 8.37 (dd, J = 7.7, 1.3 Hz 1H), 8.32 (dd, J = 7.7, 1.6Hz 1H), 8.29-8.25 (m, 2H), 8.18-8.15 (m, 1H), 8.05 (dd, J = 7.9, 1.3Hz, 1H), 8.01 (t, J = 7.8 Hz, 1H), 7.83-7.72 (m, 4H), 7.58 (t, J = 7.8Hz, 1H), 6.75(dd, J = 7.9, 1.4Hz, 2H), 6.68-6.59 (m, 2H), 6.50 (ddd, J = 7.9, 7.5, 1.5Hz, 2H)
^{13}C NMR (100 MHz, δ, ppm): 182.84, 180.94, 144.39, 141.50, 140.94, 138.30, 137.29, 136.26,134.73, 134.60, 134.28, 134.09, 134.00, 133.89, 133.59, 132.58, 131.55, 128.70, 127.91, 127.80, 127.66, 126.98, 126.94, 123.24, 121.61, 115.85, 112.30

MS^{+}:389.1065

Ant-1-Carb 1-(carbazol-9-yl)anthraquinone

Yield: from 0.53 g to 0.70 g (41%-54%), orange

^{1}H NMR (400 MHz, $CDCl_3$, δ, ppm): 8.60 (dd, J = 7.8, 1.4 Hz, 1H), 8,31 (dd, J = 7.8, 0.9 Hz, 1H), 8.21 (ddd, J = 7.6, 1.3, 0.7 Hz, 2H), 8.01 (t, J = 7.8 Hz, 1H), 7.94 (ddd, J, 7.8, 1.3, 0.4 Hz, 1H), 7.90 (dd, J = 7.8, 1.4, 1H), 7.76 (td,, J = 7.5, 1.3Hz, 1H), 7.68 (td, J = 7.5, 1.3 Hz, 1H), 7.40 – 7.27 (m, 4H), 7.03 (d, J = 7.8, 2H)
^{13}C NMR (100 MHz, δ, ppm): 182.94, 181.09, 141.13, 137.43, 137.24, 136.27, 135.03, 134.59, 134.40, 133.98, 132.61, 130.13, 128.37, 127.69, 126.98, 126.08, 123.83, 120.73, 120.19, 109.47

MS^{+}:373.1117

Ant-1-NPh2 1-(diphenylamino)anthraquinone

Yield: from 0.78 g to 0.94 g (60%-72%), bright red

^{1}H NMR (400 MHz, $CDCl_3$, δ, ppm): 8.21 (ddd, J = 7.7, 1.6, 0.5 Hz, 1H), 8.18 (dd, J = 7.6 Hz, 1H), 7.92 (ddd, J = 7.4, 1.6, 0.5Hz, 1H), 7.71-7.63 (m, 3H), 7.56 (dd, J = 8.1, 1.3Hz, 1H), 7.21 (dd, J = 8.6, 7.3 Hz, 4H), 7.06-7.03 (m, 4H), 7.00-6.95 (m, 2H)
^{13}C NMR (100 MHz, δ, ppm): 183.35, 181.00, 148.10, 147.86, 136.65, 136.48, 135.12, 134.36, 134. 24, 133.28, 132.66, 129.26, 128.27, 127.13, 126.68, 124.33, 123.08, 122.98

MS^{+}:375.1274

Table S1. Optical transitions and their oscillator strengths calculated for the studied anthraquinone derivatives; TD-B3LYP/6-311++g(d,p) method was applied.

name	transition	wavelength	osc. Strength	major contribution
anthraquinone	1	410.478	0	HOMO → LUMO (70%)
	2	375.336	0	H-3 → LUMO (68%)
	3	350.983	0	H-1 → LUMO (70%)
	4	333.321	0.173	H-3 → LUMO (69%)
Anth-1-NPh2	1	660.900	0.067	HOMO → LUMO (70%)
	2	422.349	0.063	HOMO → L+1 (66%)
	3	414.568	0.037	H-3 → LUMO (39%)
	4	380.158	0.002	H-1 → LUMO (90%)
Anth-2-NPh2	1	567.203	0.154	HOMO → LUMO (70%)
	2	406.908	0	H-3 → LUMO (65%)
	3	381.48	0.253	HOMO → L+1 (68%)
	4	373.246	0.003	H-8 → LUMO (66%)
	5	344.441	0.030	H-1 → LUMO (60%)
	6	337.677	0.167	H-2 → LUMO (52%)
Anth-1-Carb	1	645.452	0	HOMO → LUMO (70%)
	2	528.247	0	H-1 → LUMO (71%)
	3	414.651	0	H-3 → LUMO (58%)
	4	410.424	0.013	HOMO → L+1 (61%)
	1	568.999	0.085	HOMO → LUMO (70%)

Anth-2-Carb	2	485.492	0	H-1 → LUMO (71%)
	3	409.380	0	H-3 → LUMO (69%)
	4	380.695	0.109	HOMO → L+1 (67%)
Anth-1-Phenox	1	1032.261	0	HOMO → LUMO (70%)
	2	536.685	0	HOMO → L+1 (70%)
	3	460.842	0	H-1 → LUMO (68%)
	4	416.812	0	H-4 → LUMO (67%)
Anth-2-Phenox	1	875.105	0	HOMO → LUMO (71%)
	2	497.073	0	HOMO → L+1 (70%)
	3	433.665	0	H-1 → LUMO (68%)
	4	410.220	0	H-4 → LUMO (68%)

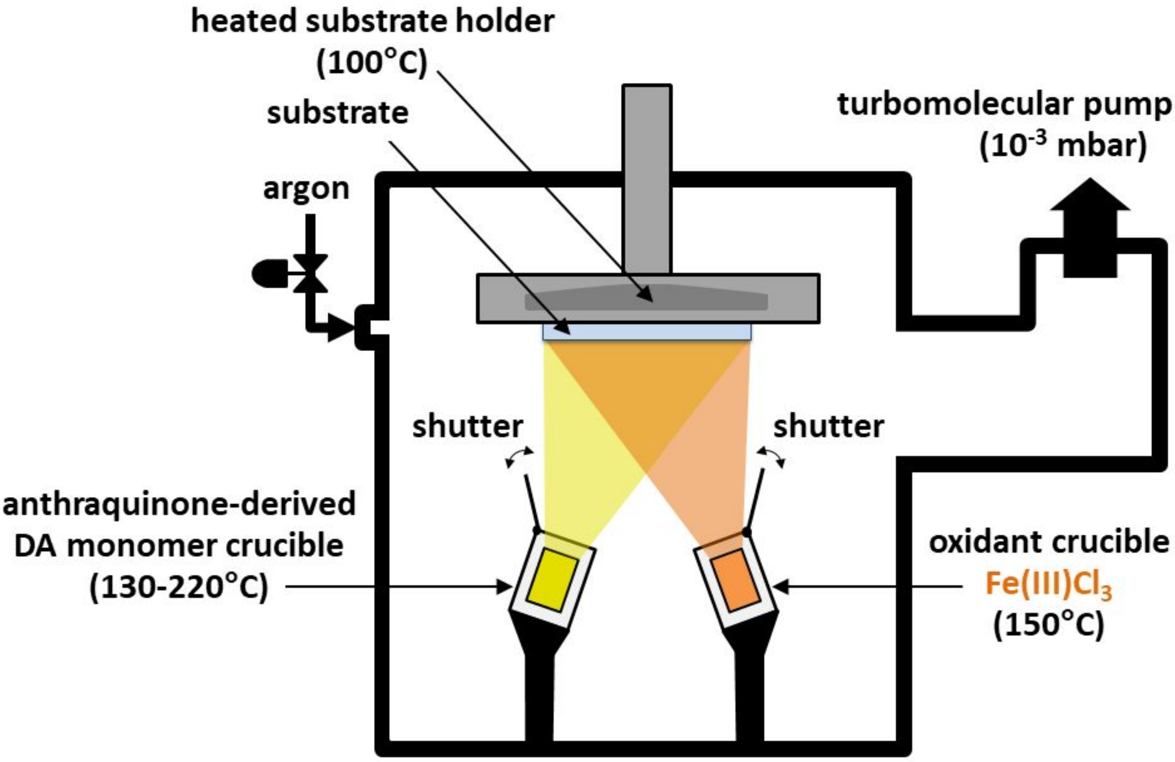

Scheme S1. Schematic of the custom-built oCVD reactor used for the preparation of the anthraquinone-derived DA polymer thin films. The two crucibles, placed at the bottom of the oCVD chamber and oriented upward towards the heated substrate holder (100°C), are heated to sublime anthraquinone-derived DA monomers (130-220°C) and the oxidant (150°C).

Table S2. Sublimation temperatures, amounts of sublimed reactants and oxidant-to-monomer molar ratios used for the preparation of the oCVD thin films and reference sublimed thin films prepared from **Anth-1-Carb**, **Anth-2-Carb**, **Anth-1-NPh2**, **Anth-2-NPh2**, **Anth-1-Phenox** and **Anth-2-Phenox**. For **Anth-2-NPh2** thin film, the oCVD reactor was fed with argon and maintained to a pressure of 10^{-3} mbar. The duration of the sublimation or oCVD reactions was set to 30 min.

	**Monomer**	**Sublimation Temperature**	**Sublimed Amount**	**Oxidant**	**Sublimation Temperature**	**Sublimed Amount**	**Oxidant to Monomer Ratio**
**s(Anth-1-Carb)**	**Anth-1-Carb**	220°C	48 mmol	-			0
**s(Anth-2-Carb)**	**Anth-2-Carb**	180°C	35 mmol	-			0
**s(Anth-1-NPh2)**	**Anth-1-NPh2**	155°C	101 mmol	-			0
**s(Anth-2-NPh2)**	**Anth-2-NPh2**	205°C	28 mmol	-			0
**p(Anth-2-NPh2)**	**Anth-2-NPh2**	205°C	31 mmol	$FeCl_3$	150°C	885 mmol	28
**s(Anth-1-Phenox)**	**Anth-1-Phenox**	130°C	28 mmol	-			0
**s(Anth-2-Phenox)**	**Anth-2-Phenox**	205°C	53 mmol	-			0

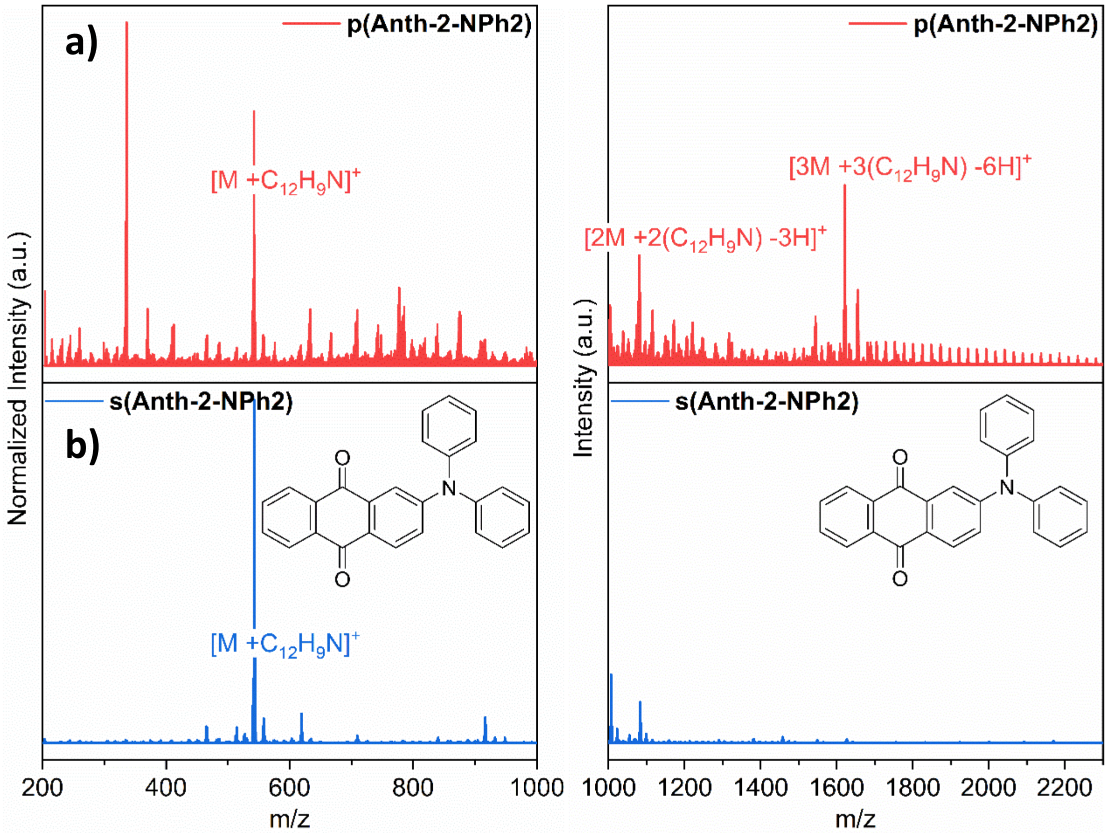

Figure S1. LDI-HRMS spectra in the mass ranges m/z 200−1000 (left) and m/z 1000-2500 of the oCVD **p(Anth-2-NPh2)** (**S1a**) thin film prepared from **Anth-2-NPh2** and $FeCl_3$ (solid red) and the reference sublimed **s(Anth-2-NPh2)** (**S1b**) thin film prepared from **Anth-2-NPh2**.

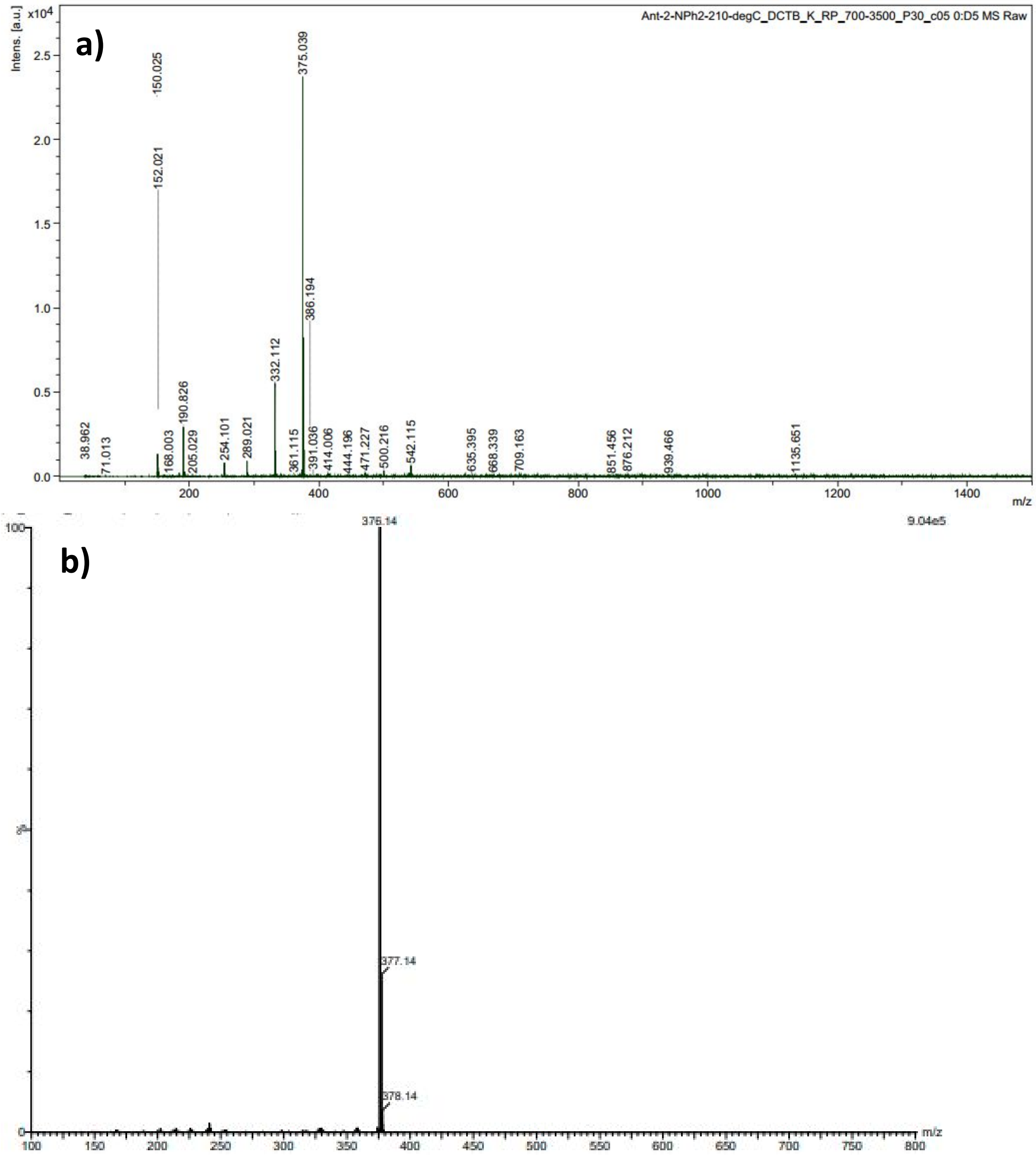

Figure S2:**a)** APCI-HRMS spectra in the mass ranges m/z 0−1500 and **b)** EI-HRMS spectra in the mass ranges m/z 100−800 of **Anth-2-NPh2.**

a) **b)**

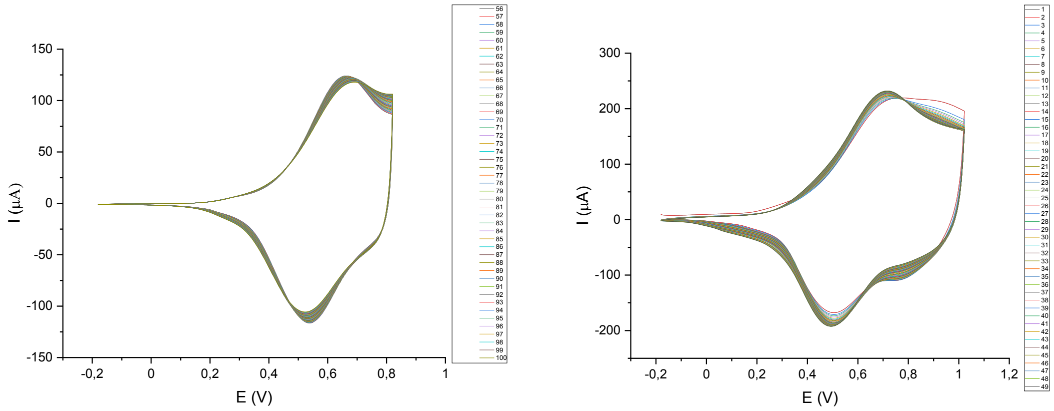

Figure S3 Cyclic voltammograms of a thin film of p(Anth-2-NPh2) registered for 100 consecutive scans. Electrolyte 0,1M Bu_4NBF_4 in CH_3CN; scan rate **50**mV/s; potential vs Fc^+/Fc.

a) Potential range of scans: from -0.2V to 0.8 V;
b) Potential range of scans: from -0.2V to 1.0 V;

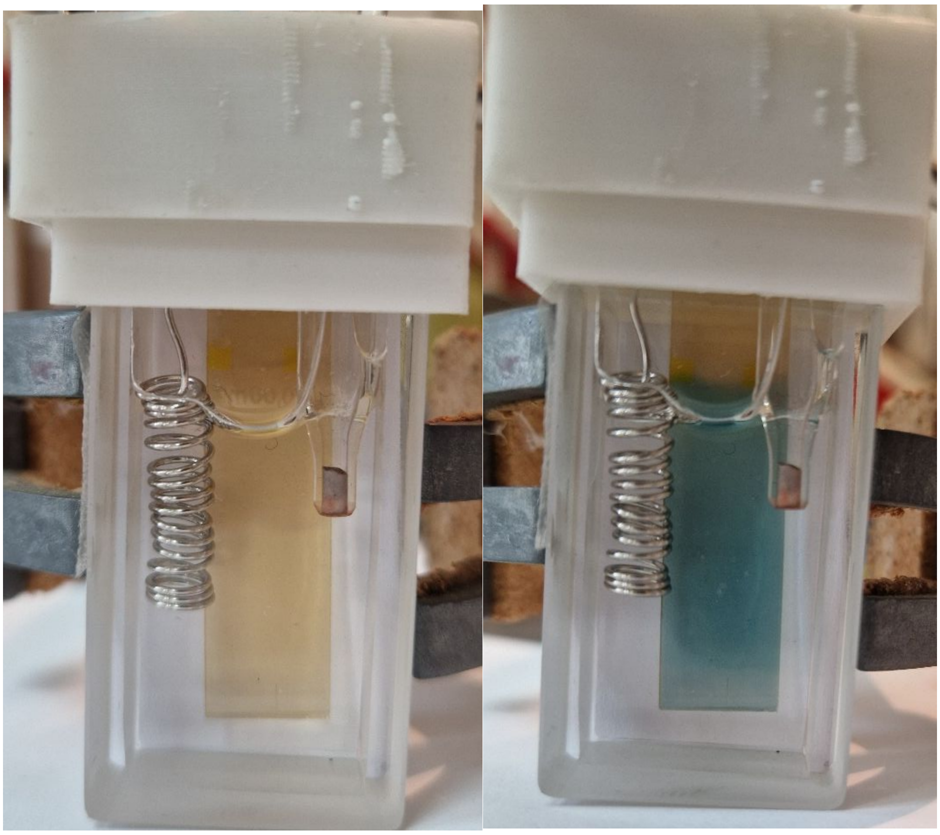

a) **b)**

Figure S4 Thin layer of p(Anth-2-NPh2): a) in its neutral state (E=-0.18 V); b) in its fully oxidized state (E= 0.92 V).

Table S3. Raman frequencies (in cm^{-1}) of the main vibrational modes of **Anth-2-NPh2,** Anthraquinone (**AQ**) and **p(Anth-2-NPh2)** in its neutral and oxidation state.

**Monomer**		**AQ**	**p(Anth-2-NPh2)** **Potential vs. Fc/Fc**				
**Anth-2-NPh2**		**anth**	-0.08V		+0.62V	+0.92V	Assignments
Exptl	Calcd	Exptl	Exptl	Calcd	Exptl	Exptl	
1657	1653						AQ C=O stretching asym
1645	1648	1665	1655	1651	1670		AQ C=O stretching sym
1608	1610		1604	1610	1600	1612	Aromatic ring C-C stretch
1585	1582	1596	1586	1583			AQ Aromatic C-C stretch
						1578	Quinoid C=C stretch (dication)
					1569		Semi-quinonoïd C=C stretch (radical cation)
					1521	1528	quinonoïd ring, C=C stretch
1496	1512		1489	1512	1489-1479	1482	AQ C-C stretch + CH bend
			1443	---	1442	1433	C-N°⁺ stretch (semiquinone radical cation)
			1420	---	1420	1420	C-N⁺ stretch (quinoid diiminium dication)
			1341	1338	1337	1356-1330	C-N⁺ stretch (quinoid diiminium dication)
1336	1336	1318					AQ CC Strech
					1295-1318		C-N°⁺ stretch (semiquinone radical cation)
1283	1283		1288	1283			Aromatic CH bend
					1206	1207	C-H deformation (quinoid diiminium dication)
						1173	C-H deformation (quinoid diiminium dication)
1195	1193	1178	1181	1192	1185		C-H deformation
1145	1129	1148	1145	1128	1146		AQ CH bend
1031	1013	1030					AQ CC stretch
1000	1009				996		Aromatic ring deformation (para)
987	985		986	986	986		Aromatic ring deformatiAon (para)
					930-900	927-890	quinonoïd ring deformation
